# Melatonin Improves In Vitro Development of Vitrified-Warmed Mouse Germinal Vesicle Oocytes Potentially via Modulation of Spindle Assembly Checkpoint-Related Genes

**DOI:** 10.3390/cells8091009

**Published:** 2019-08-30

**Authors:** Zhenzheng Wu, Bo Pan, Izhar Hyder Qazi, Haoxuan Yang, Shichao Guo, Jingyu Yang, Yan Zhang, Changjun Zeng, Ming Zhang, Hongbing Han, Qingyong Meng, Guangbin Zhou

**Affiliations:** 1 Farm Animal Genetic Resources Exploration and Innovation Key Laboratory of Sichuan Province, College of Animal Science and Technology, Sichuan Agricultural University, Chengdu 611130, China; 2 Department of Veterinary Anatomy & Histology, Shaheed Benazir Bhutto University of Veterinary and Animal Sciences, Sakrand 67210, Sindh, Pakistan; 3 National Engineering Laboratory for Animal Breeding, Key Laboratory of Animal Genetics and Breeding of the Ministry of Agriculture, Beijing Key Laboratory for Animal Genetic Improvement, College of Animal Science and Technology, China Agricultural University, Beijing 100193, China; 4 State Key Laboratory of AgroBiotechnology, China Agricultural University, Beijing 100193, China

**Keywords:** melatonin, oocyte vitrification, redox homeostasis, mitochondrial membrane potential, ATP content, spindle assembly checkpoint, mouse

## Abstract

The present study aimed to investigate the effect of melatonin (MT) supplementation on in vitro maturation of vitrified mouse germinal vesicle (GV) oocytes. The fresh oocytes were randomly divided into three groups: untreated (control), or vitrified by open-pulled straw method without (vitrification group) or with MT supplementation (vitrification + MT group). After warming, oocytes were cultured in vitro, then the reactive oxygen species (ROS) and glutathione (GSH) levels, mitochondrial membrane potential, ATP levels, spindle morphology, mRNA expression of spindle assembly checkpoint (SAC)-related genes (*Mps1*, *BubR1*, *Mad1*, *Mad2*), and their subsequent developmental potential in vitro were evaluated. The results showed that vitrification/warming procedures significantly decreased the percentage of GV oocytes developed to metaphase II (MII) stage, the mitochondrial membrane potential, ATP content, and GSH levels, remarkably increased the ROS levels, and significantly impaired the spindle morphology. The expressions of SAC-related genes were also altered in vitrified oocytes. However, when 10^−7^ mol/L MT was administered during the whole length of the experiment, the percentage of GV oocytes matured to MII stage was significantly increased, and the other indicators were also significantly improved and almost recovered to the normal levels relative to the control. Thus, we speculate that MT might regulate the mitochondrial membrane potential, ATP content, ROS, GSH, and expression of SAC-related genes, potentially increasing the in vitro maturation of vitrified-warmed mouse GV oocytes.

## 1. Introduction

As an artificial assisted reproductive technology, oocyte cryopreservation has considerable application in medical, animal, and agricultural sciences [1,2,3,4]. It serves as an important and promising tool for conservation and propagation of germplasm and improving the reproductive efficiency in several mammalian species [5]. Particularly in medical sciences, it has provided substantial relief to women suffering from reproductive insufficiencies such as ovarian cancer and premature ovarian failure [6,7,8]. However, despite the significant and tangible advances made in the area of cryopreservation of mammalian germplasm, the developmental potential of oocytes and embryos following vitrification/warming still poses considerable obstacles [5,9]. In particular, the molecular mechanisms implicated in impaired/reduced development of germinal vesicle (GV) stage oocytes following cryopreservation are poorly understood and require thorough elucidation.

Although, in vitro fertilization of cryopreserved GV oocytes could successfully yield offspring [10,11,12,13], the resultant survival ratio, fertilization rates, and developmental competence are considerably lower [10,14,15,16,17]. In mouse, blastocyst rate of vitrified-warmed GV oocytes was 17–42.9% after in vitro fertilization [13,14,15,18,19]. In pig, the blastocyst rate of vitrified-warmed GV oocytes following in vitro maturation and parthenogenetic activation was reduced to 9.6–35.6% [20,21]. Such perturbations in the developmental competence of mammalian oocytes could be attributed to the impaired redox status, increased levels of reactive oxygen species (ROS) [1], mitochondrial dysfunction [22], abnormal spindle configuration [23], and the altered expression of important regulatory genes [1,24].

Similarly, mitochondrial activity and ATP concentrations are also decreased in GV oocytes following vitrification-warming procedures [25,26], impairing the microtubule function [27,28]. In mouse, in vitro maturation of vitrified GV oocytes to metaphase II (MII) oocytes resulted in the decreased mitochondrial activity [29], and significantly impaired the spindle configuration [17]. In pig, the percentage of normal spindles was significantly decreased in MII oocytes derived from vitrified-warmed GV oocytes [30]. In bovine, when GV oocytes were vitrified-warmed and in vitro maturated to MII stage, the mitochondrial distribution was impaired, subsequently affecting their developmental potential [31]. Therefore, this evidence indicates that redox balance, mitochondrial function, and adequate supply of ATP are critical for maintaining the normal spindle configuration, and could play an important role in ameliorating the developmental potential of vitrified-warmed GV oocytes.

In addition, vitrification-warming procedures are believed to perturb the relative expression of important genes in immature oocytes and result in reduced developmental potential. In goat, following in vitro maturation of vitrified-warmed immature oocytes, the relative mRNA expression of *GDF9*, *BMP15*, *TGFBR1*, and *BAX* was increased, and the relative mRNA expression of *BMPR2*, *BCL2*, and *P53* was decreased [32]. Besides, in mouse model, the relative expressions of *Dcp1a*, *Dcp2*, and *Oct4* were decreased after 0–2 h in vitro culture of vitrified-warmed mature oocytes [24]. During meiosis of mammalian oocytes, the spindle assembly checkpoint (SAC) ensures that chromosome segregation occurs only after two ends of spindle have been correctly ligated [33,34]. The SAC involves the MAD (mitotic arrest deficient) proteins MAD1, MAD2, BUBR1 (MAD3), and BUB1 [35]. MPS1 (multipolar spindle-1), a dual specificity kinase, is a core SAC protein that regulates kinetochore recruitment of other SAC proteins [36]. Deletion of SAC-associated proteins affects oocyte meiosis and leads to an impaired segregation of sister chromosomes, contributing to the reduced developmental potential of oocytes [37,38]. However, it is still unclear whether the expression of SAC-related genes is impaired following vitrification-warming of mouse oocytes. Besides, its relationship with oocyte developmental potential is also largely unexplored.

The addition of melatonin (MT) to human [39], rat [40], porcine [41], and bovine [42] oocyte culture systems has been reported. It could promote the in vitro maturation of oocytes either through indirect antioxidant action [41,43,44,45], or by directly reducing the oxidative stress [46,47,48] during in vitro culture. Besides, MT has been implicated in playing an important role in the development of vitrified-warmed oocytes, potentially leading to their improved in vitro maturation [49]. In our recent reports, we have demonstrated that 10^−9^ mol/L MT could ameliorate blastocyst development rate (16.19–33.61% vs. 41.6–57.14%) of vitrified mouse MII oocytes after parthenogenetic activation [1,24]. This improved developmental potential of vitrified-warmed oocytes is associated with the antioxidant activity of MT [1,24], increased mitochondrial membrane potential levels [31] and the induced promotion of cell cycle progression from G1 to S via modulation of expression of cell cycle-related genes [1]. However, apparently it seems that these ameliorative effects of MT are probably concentration dependent [24] and could vary at different concentrations of MT and developmental stage of oocytes. Nevertheless, the optimal concentration of MT for ameliorating the in vitro maturation of vitrified-warmed mouse GV oocytes and its potential mechanism of action still remain largely unclear, and require further elucidation.

Therefore, the first phase of this research was aimed to screen the optimal concentrations of MT (10^−9^, 10^−7^, 10^−5^, and 10^−3^ mol/L) to be used subsequently in the whole procedures used for vitrification-warming and in vitro maturation of mouse GV oocytes. Next, the effects of MT on in vitro maturation of vitrified-warmed GV oocytes and its possible mechanisms of action were studied. In subsequent experiments, ROS and glutathione (GSH) levels in oocytes, mitochondrial membrane potential, and ATP levels, spindle morphology, and expression of SAC-related genes (*Mps1*, *BubR1*, *Mad1*, and *Mad2*) were studied in vitrified-warmed and in vitro matured mouse GV oocytes. Our experimental results provide a reasonable theoretical reference for further improving the in vitro development of vitrified-warmed oocytes.

## 2. Materials and Methods

Unless otherwise indicated, all chemicals were purchased from Sigma-Aldrich (St. Louis, MO, USA). All experimental procedures were carried out in strict accordance to the regulations of the animal ethical and welfare committee (AEWC) of Sichuan Agricultural University China (approval code: AEWC2016, 6 January 2016).

### 2.1. Oocyte Collection

Six weeks old outbred female ICR mice (*n* = 110) were obtained from Dashuo Company, Chengdu, China, and kept in sterilized cages under standard housing conditions as described in our previous study [1]. The ambient temperature was kept 18–22 °C, and the humidity was maintained 50–70%. After a two-week adaptation period, female mice were induced to superovulate by an initial intraperitoneal injection of 10 IU equine chorionic gonadotropin (PMSG, NingBo second hormone factory, Ningbo, China), and after 44–48 h ovaries were removed and placed in a 37 °C M2 solution. GV oocytes (with 2–3 layers of granulosa cells) were collected from ovarian follicles punctured by a syringe needle. During whole experiment, *c.* 3800 GV oocytes were collected. From these, 3645 GV oocytes were used in different assays during the entire course of this study (break up details are given in the results section). For subsequent experiments, all pooled GV oocytes were randomly divided into three groups: fresh group (control), vitrification group (without MT), and vitrification + MT group.

### 2.2. Experimental Groups and Screening of Optimal Concentration of Melatonin

For screening of optimal concentration of MT to be used in further experiments, GV oocytes in vitrification + MT group were further divided into four independent groups and treated with different concentrations (10^−9^, 10^−7^, 10^−5^, 10^−3^ mol/L) of MT added in all media (10% ethylene glycol (EG) + 10% dimethyl sulfoxide (DMSO), EDFS30, 0.5 mol/L sucrose and M16) used in vitrification-warming procedures and in vitro maturation steps. Oocytes in vitrification group did not receive any MT treatment, and a fresh group without MT was kept as a control. The in vitro development rate (i.e., maturation of GV oocytes to MII stage) of GV oocytes was evaluated at this stage. The concentration of MT (10^−7^ mol/L) at which highest devolvement rate was observed was then adopted for further experiments.

### 2.3. Oocyte Vitrification and Warming

Oocytes were vitrified using an open-pulled straw (OPS) method. For this purpose, OPS were prepared as described previously [50]. In brief, the straws (250 µL; IMV, L′Aigle, France) were heat-softened, pulled manually, and straws of approximately 3 cm in length, 0.10 mm inner diameter, and 0.15 mm outer diameter were obtained.

Vitrification-warming procedures were carried out as per our laboratory practice [1]. Briefly, for vitrification, oocytes were first equilibrated in 10% EG + 10% DMSO for 30 s, then loaded into the narrow end of an OPS with EDFS30 solution comprising of Dulbecco’s phosphate-buffered saline (DPBS) medium containing 300 g/L Ficoll, 0.5 mol/L sucrose, and 20% fetal bovine serum (FBS), 15% (*v*/*v*) EG and 15% (*v*/*v*) DMSO, with exposure for 25 s. Finally, the straws containing oocytes (eight oocytes per OPS) were rapidly plunged into liquid nitrogen. For warming, oocytes were rinsed in 0.5 mol/L sucrose for 5 min, then washed three times in M2 medium and incubated in an incubator (Thermo Electron Corporation, Marietta, OH, USA) at 37.5 °C with 5% CO_2_ in air for 1 h in M16 medium. All manipulations were performed at 37 °C on a warming stage fixed onto the stereomicroscope, and the ambient atmosphere was air-conditioned at a temperature of 25 ± 0.5 °C.

### 2.4. Oocyte Culture and In Vitro Maturation

Initially, oocytes in all groups were allowed to recover in a M16 medium, and then incubated (5% CO_2_, 37.5 °C, 100% humidity) for 1, 5, 9, 13 h to yield GV, GVBD, MI, and MII oocytes, respectively.

### 2.5. Detection of Mitochondrial Membrane Potential

For detection of mitochondrial membrane potential, GV, MI, and MII oocytes from each group were collected and assayed using JC-1 according to the manufacturer’s guidelines (JC-1; Beijing Solarbio Science & Technology Co., Ltd., Beijing, China). Briefly, the JC-1 probe was diluted to a working concentration of 10 µg/mL with M2 solution before use, and oocytes were transferred to the staining solution, and stained at 37 °C for 15 min. After staining, oocytes were washed three times (3 × 5 min) with M2 solution without JC-1 probe. Finally, oocytes were placed on a clean glass slide, and photographed under a fluorescence microscope (BX53, Olympus, Tokyo, Japan). The fluorescence images were recorded as TIFF files using a built-in camera. The intensity of red and green fluorescence in each oocyte was measured using Image J software (version 1.48; National Institutes of Health, Bethesda, MD). The ratio of red fluorescence to green fluorescence was recorded as mitochondrial membrane potential (∆ψm) of oocytes. For fluorescence microscopy, the excitation and emission conditions were as follows: JC-1 monomer (excitation: 514 nm; emission: 529 nm) and JC-1 polymer/JC-aggregates (excitation: 585 nm; emission: 590 nm). Fluorescein isothiocyanate (FITC, green) and rhodamine isothiocyanate (RITC, red) channels were used to observe the mitochondrial membrane potential in oocytes.

### 2.6. Detection of ATP Content in Oocytes

GV, MI, and MII oocytes from each group were collected and processed for detection of ATP content. For this purpose, oocytes were initially washed three times with M2 and added to an Eppendorf tube containing 20 μL of ATP lysate for ATP detection (groups of 10 oocytes). ATP levels were determined according to the manufacturer’s instructions (A095-2, Nanjing Jiancheng Bioengineering Institute, Nanjing, China). Briefly, 100 μL of enzyme working solution was added to a 96-well plate and kept at room temperature for 5 min. Then oocytes containing 20 μL of ATP lysate were transferred to each well of a 96-well plate (containing enzyme working solution) and mixed quickly with a pipette. At least after 2 s interval, ATP detection assay was completed and ATP levels were measured using a multi-plate reader containing chemiluminescence. Sample ATP concentration was calculated using a standard curve generated from nine ATP gradient concentrations ranging from 0 mol/L to 2 μmol/L.

### 2.7. Spindle Morphology and Classification

For evaluation of spindle morphology and classification, mouse MII oocytes were fixed in 4% (*w*/*v*) paraformaldehyde and then permeabilized in permeate (PBS containing 1% Triton X-100 (*v*/*v*) for 1 h at room temperature. After blocking of oocytes with 1% BSA for 1 h at room temperature, they were stained with FITC-anti-α-tubulin antibody (Sigma, F2168) at a dilution of 1:2000 for 1 h at room temperature, and washed three times for 5 min each in the wash buffer (PBS containing 0.01% Triton X-100 and 0.1% Tween 20). DNA was stained with DAPI (Vector Laboratories Inc., Burlingame, CA, USA). Oocytes were finally placed on a clean glass slide and slides were scanned under a confocal microscope (A1SI, Nikon, Tokyo, Japan).

The spindle configuration was graded as described in Tamura et al. [28]. In short, Grade 0 = severely diminished spindle, i.e., less than 50% of the normal spindle in size. Grade 1 = mildly diminished spindle, i.e., larger than 50% of the normal spindle. Grade 2 = severely increased spindle, i.e., larger than the normal spindle in size. Grade 3 = equivalent to the normal spindle in size and shape.

### 2.8. Measurement of Intracellular ROS and GSH Levels

The levels of ROS and GSH in GV, MI, and MII oocytes of each group were detected according to our laboratory’s protocol as described previously [1]. Briefly, for quantification of intracellular ROS levels, oocytes from each group were incubated (in dark) in M2 supplemented with 100 μM 2, 7-dichlorodihydrofluorescein diacetate (H_2_DCFDA, Invitrogen, Carlsbad, CA, USA) for 20 min at 37 °C, washed three times in M2 medium containing 3 mg/mL bovine serum albumin, and then put into 8 μL droplets of fluorescent mounting medium with DAPI (Vector Laboratories Inc., Burlingame, CA, USA) on a slide, then covered with a cover slip. Fluorescence was measured under an epifluorescence microscope (BX53, Olympus, Tokyo, Japan) with a filter at 460-nm excitation, and fluorescence images were recorded as TIFF files using a built-in camera. The recorded fluorescence intensities were quantified using Image J software (version 1.48; National Institutes of Health, Bethesda, MD, USA) after deducting the background value. The level of GSH in each oocyte was measured with 10 μM 4-chloromethyl-6.8-difluoro-7-hydroxycoumarin (Cell-Tracker Blue, Invitrogen, Carlsbad, CA, USA) with a filter at 370-nm excitation. The experimental procedure was the same as the ROS measurement described above.

### 2.9. Quantitative Polymerase Chain Reaction (Q-PCR)

In each group, total complementary DNA (cDNA) was obtained from oocytes (*n* = 20–25) at GV, GVBD, MI, and MII stages using TransScript-Uni Cell to cDNA Synthesis SuperMix for Q-PCR (TransGen Biotech, Beijing, China). Then, cDNA was quantified by Q-PCR using TransStart Tip Green qPCR SuperMix (TransGen Biotech, Beijing, China) on a CFX Connect Real-Time Detection System (Bio-Rad, Hercules, CA, USA) under standard conditions. Three replicates were performed for this assay, and the relative mRNA expression levels were obtained using the 2^−∆∆Ct^ method [51]. *Gapdh* was used as a reference gene for normalization [52,53,54]. The primer information is detailed in Table 1.

### 2.10. Statistical Analyses

All experiments were replicated at least three times. Statistical analyses were performed using one-way ANOVA followed by a post-hoc Fisher’s least significant difference (LSD) test using SPSS statistical software (v. 20; IBM, Chicago, IL, USA). A *p* value < 0.05 was considered as statistically significant.

## 3. Results

### 3.1. Effect of Different Concentrations of Melatonin on In Vitro Maturation of Vitrified-Warmed Mouse GV Oocytes

As shown in Table 2, following vitrification-warming, the in vitro development rate of mouse GV oocytes (ratio of GVBD, MI, and MII) was significantly lower (*p* < 0.05) in vitrification group than that of the control (fresh group). However, in vitrification + MT groups, the ratio of GV oocytes to MII was significantly higher (*p* < 0.05) only in 10^−7^ mol/L MT-treated group compared to the vitrification group (72.67% vs. 61.30%). Besides, the development rates of GV oocytes in other MT-treated groups (10^−9^, 10^−5^, and 10^−3^ mol/L) were significantly lower (*p* < 0.05) than that of the vitrification group (19.39–37.92% vs. 61.30%). Therefore, based on foregoing observations, we resolved that addition of 10^−7^ mol/L MT might be an optimal concentration for ameliorating the in vitro developmental potential of vitrified GV mouse oocytes, and hence 10^−7^ mol/L MT was selected for subsequent experiments.

### 3.2. Melatonin Supplementation Ameliorates ROS Levels in Vitrified-Warmed Mouse GV Oocytes and Their In Vitro-Derived MI and MII Stage Oocytes

**Figure 1:**
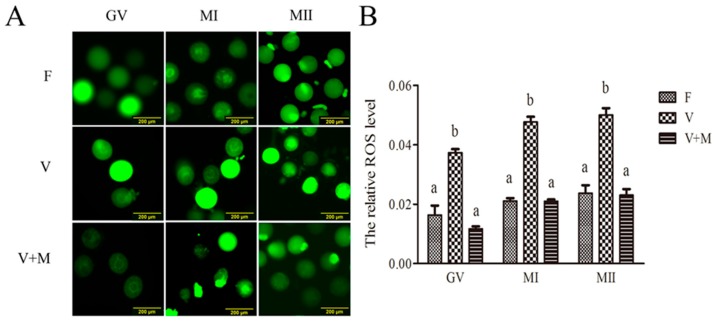
Effects of melatonin (MT) supplementation on reactive oxygen species (ROS) levels in vitrified-warmed mouse GV oocytes during in vitro maturation. (**A**): Representative fluorescence images depicting ROS intensities in vitrified-warmed mouse oocytes at different stages of development (GV, MI, and MII). The oocytes were treated with H_2_DCFDA for detection of ROS levels (green). Fresh control (F), vitrification (V), and vitrification + MT (V + M). Scale bars = 200 μm. (**B**): Quantification of relative levels of ROS in oocytes. Fluorescence intensities of ROS levels were measured using ImageJ software. Data are presented as mean ± SEM of three independent experiments. Total number of GV oocytes used in this assay *n* = 338 (F: *n* = 108; V: *n* = 119; V + M: *n* = 111). Different superscripts (a and b) indicate significant difference (*p* < 0.05) between groups at the same time point (at corresponding developmental stages).

As depicted in Figure 1, following vitrification-warming, ROS levels were significantly increased (*p* < 0.05) in GV oocytes compared to the fresh group (control). Similarly, ROS levels in oocytes developed to MI and MII stages were also significantly higher (*p* < 0.05) than those of the corresponding fresh group. However, in vitrification + MT (10^−7^ mol/L) group, ROS levels in oocytes (GV, MI, and MII) were significantly lower (*p* < 0.05) than that of the corresponding vitrified group. Intriguingly, no significant difference (*p* > 0.05) was observed between MT-treated (10^−7^ mol/L) and fresh groups. These observations clearly indicate that addition of MT during vitrification-warming and in vitro maturation procedures can scavenge excessive ROS in vitrified-warmed mouse GV, MI, and MII oocytes.

### 3.3. Melatonin Supplementation Ameliorates GSH Levels in Vitrified-Warmed Mouse GV Oocytes and Their In Vitro-Derived MI and MII Stage Oocytes

**Figure 2:**
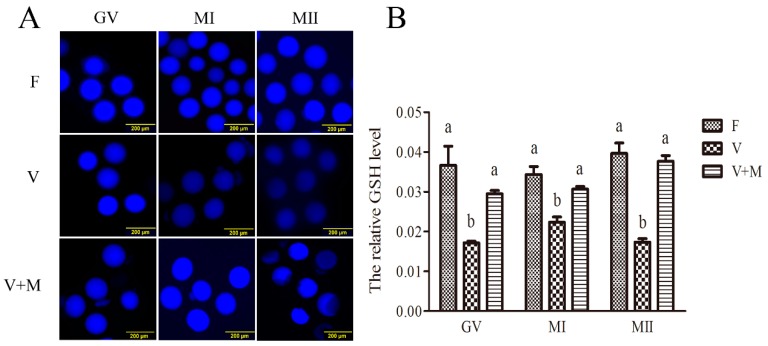
Effects of melatonin (MT) on GSH levels in vitrified-warmed mouse GV oocytes during in vitro maturation. (**A**): Representative fluorescence images depicting GSH levels in vitrified-warmed mouse oocytes at different stages of development (GV, MI, and MII). The oocytes were treated with CMF2 H for detection of GSH levels (blue). Fresh control (F), vitrification (V), and vitrification + MT (V + M). Scale bars = 200 μm. (**B**): Quantification of relative levels of GSH in oocytes. Fluorescence intensities of GSH levels were measured with ImageJ software. Data are presented as mean ± SEM of three independent experiments. Total number of GV oocytes used in this assay *n* = 352 (F: *n* = 116; V: *n* = 115; V + M: *n* = 121). Different superscripts (a and b) indicate significant difference (*p* < 0.05) between groups at the same time point (at corresponding developmental stages).

As shown in Figure 2, following vitrification-warming, GSH levels in mouse GV, MI, and MII oocytes were significantly decreased (*p* < 0.05) compared to the corresponding fresh group (control). However, in vitrification + MT (10^−7^ mol/L) group, significantly higher (*p* < 0.05) GSH levels were observed in GV, MI, and MII oocytes compared to those in the corresponding vitrified group. Interestingly, comparable (*p* > 0.05) GSH levels were also observed between vitrification + MT (10^−7^ mol/L) and the control groups.

### 3.4. Effect of Melatonin Supplementation on Mitochondrial Membrane Potential in Vitrified-Warmed Mouse GV Oocytes and Their In Vitro-Derived MI and MII Stage Oocytes

**Figure 3:**
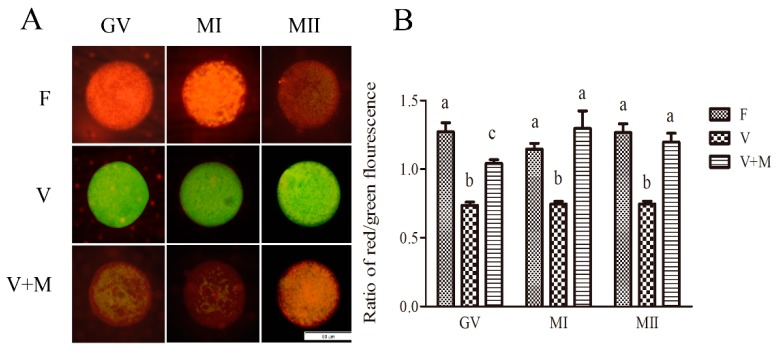
Effects of melatonin (MT) on mitochondrial membrane potential in vitrified-warmed mouse GV oocytes during in vitro maturation. (**A**): Representative fluorescence images depicting the mitochondrial membrane potential levels in vitrified-warmed mouse oocytes at different stages of development (GV, MI, and MII); (oocytes stained with JC-1). Fresh control (F), vitrification (V), and vitrification + MT (V + M). Scale bar = 50 μm. (**B**): Mitochondrial membrane potential levels in vitrified-warmed mouse oocytes. Fluorescence intensities were measured using ImageJ software. In total, *n* = 348 GV oocytes were used in this assay (F: *n* = 104; V: *n* = 138; V + M: *n* = 106). Data are presented as mean ± SEM of three independent experiments. Different superscripts (a, b, and c) indicate significant difference (*p* < 0.05) between groups at the same time point (at corresponding developmental stages).

As shown in Figure 3, following vitrification-warming and in vitro maturation, the mitochondrial membrane potential in mouse GV, MI, and MII oocytes was significantly decreased (*p* < 0.05) in vitrification group compared to the fresh group (control). Whereas in vitrification + MT (10^−7^ mol/L) group, the mitochondrial membrane potential was significantly increased (*p* < 0.05) in GV, MI, and MII oocytes compared to the vitrified group. Meanwhile, when compared to the control, the mitochondrial membrane potential in MI and MII oocytes showed no significant difference. In general, these results corroborate that addition of 10^−7^ mol/L MT could ameliorate the mitochondrial membrane potential in vitrified-warmed mouse GV oocytes during their developmental stages.

### 3.5. Effect of Melatonin Supplementation on ATP Levels in Vitrified-Warmed Mouse GV Oocytes and Their Resultant MI and MII Oocytes

**Figure 4:**
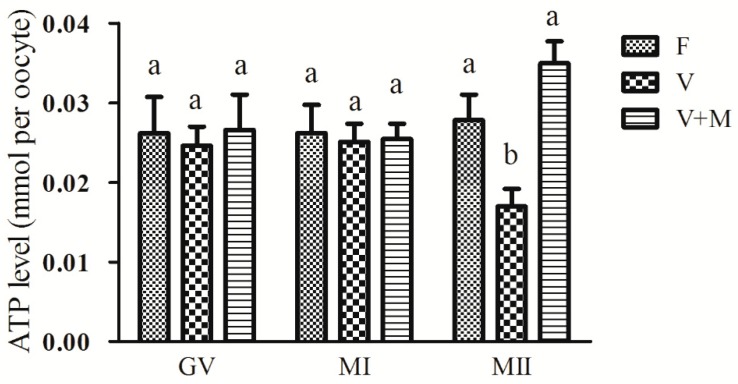
Effects of melatonin (MT) on ATP content in vitrified-warmed GV-stage mouse oocytes during in vitro maturation. ATP contents were evaluated in vitrified-warmed mouse oocytes at different stages of development (GV, MI, and MII). Fresh control (F), vitrification (V), and vitrification + MT (V + M). Total number of GV oocytes used in this assay *n* = 335 (F: *n*= 103; V: *n* = 121; V + M: *n* = 111). Data are presented as mean ± SEM of three independent experiments. Different superscripts (a and b) indicate significant difference (*p* < 0.05) between groups at the same time point (at corresponding developmental stage).

As shown in Figure 4, no significant decrease (*p* > 0.05) was observed in ATP content in vitrified-warmed mouse GV oocytes compared to the fresh group (control). Similarly, no significant difference (*p* > 0.05) in ATP content was observed in MI stage oocytes, however, a significantly lower (*p* < 0.05) ATP content was observed in MII stage oocytes compared to the corresponding control group. Intriguingly, following addition of 10^−7^ mol/L MT, ATP content was significantly increased in MII oocytes in vitro matured from vitrified-warmed GV oocytes compared to the corresponding counterparts in the vitrification group. Of note, ATP content in MII oocytes in vitrification + MT (10^−7^ mol/L) group corroborated to those in the control group.

### 3.6. Effect of Melatonin Supplementation on Spindle Configuration of MII Oocytes Derived from the Vitrified-Warmed Mouse GV Oocytes

**Figure 5:**
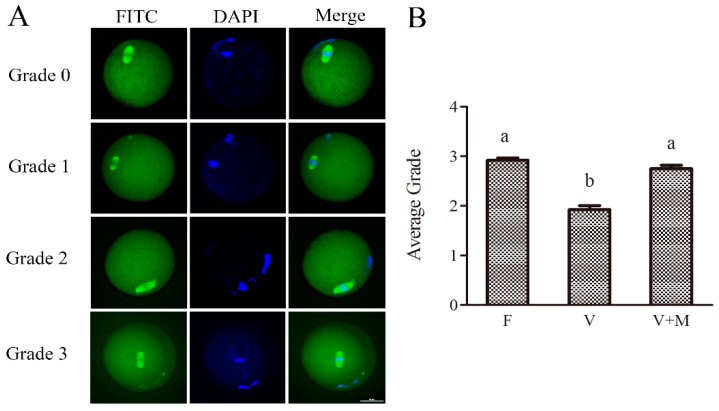
Effects of melatonin (MT) on spindle morphology of vitrified-warmed GV mouse oocytes during in vitro maturation. Spindle morphology scores were assessed in GV oocytes maturing to MII stage. (**A**): Grades of spindle morphology. Fresh control (F), vitrification (V), and vitrification + MT (V + M). Grade 0 = severely diminished spindle, i.e., that is less than 50% of the normal spindle in size. Grade 1 = mildly diminished spindle, i.e., larger than 50% of the normal spindle. Grade 2 = severely increased spindle i.e., larger than the normal spindle in size. Grade 3 = equivalent to the normal spindle in size and shape. Scale bar = 20 μm. (**B**): Average grade of spindle morphology in different groups. In total, GV oocytes were used in this assay *n* = 220 (F: *n* = 58; V: *n* = 87; V + M: *n* = 75). Data are presented as mean ± SEM of three independent experiments. Values with different superscripts (a and b) are significantly different (*p* < 0.05).

The results of spindle morphology evaluation are depicted in Figure 5. Briefly, the average score of spindle morphology in MII stage oocyte matured from fresh GV stage oocytes was observed to be grade 3. However, following vitrification-warming, when GV oocytes were in vitro cultured and matured to MII oocytes, the average grade of spindle morphology of oocytes in MT-treated (10^−7^ mol/L) group was significantly higher (2.74 vs. 1.99; *p* < 0.05) compared to the vitrified group. Meanwhile, no significant difference was observed in the average score of spindle morphology between MT-treated (10^−7^ mol/L) and the fresh (control) groups, highlighting that MT supplementation could improve the spindle morphology in vitrified-warmed oocytes during their in vitro maturation and development.

### 3.7. Effect of Melatonin on Expression of SAC-Related Genes in Vitrified-Warmed mouse GV Oocytes and Their In Vitro-Derived Oocytes at GVBD, MI, and MII Stages

**Figure 6:**
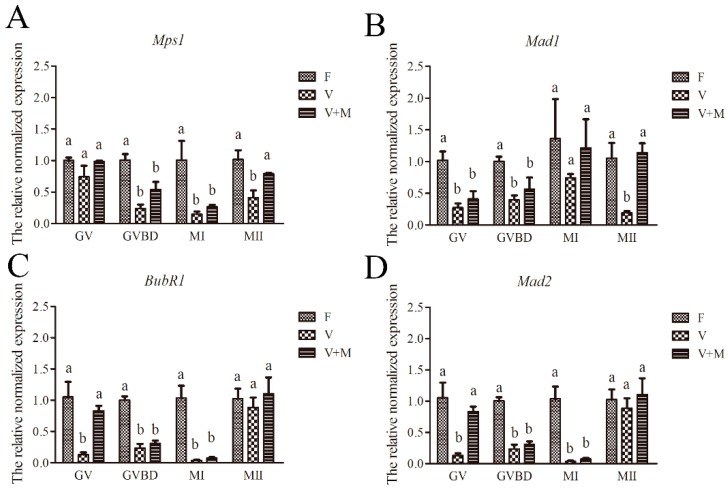
Effects of melatonin (MT) on relative mRNA expression of spindle assembly checkpoint-related (SAC) genes in vitrified-warmed GV mouse oocytes during in vitro maturation. Data are presented as mean ± SEM. The experiment was replicated at least three times. In total, *n* = 924 GV oocytes were used for mRNA expression analysis (F: *n* = 302; V: *n* = 323; V + M: *n* = 299). Different superscripts (**A**–**D**) indicate significant difference (*p* < 0.05) between groups at the same time point (corresponding developmental stages). F: fresh control oocytes without MT. V: vitrified MII oocytes without MT. V + M: vitrified MII oocytes treated with 10^−7^ mol/L MT. Different superscripts (a and b) indicate significant difference (*p* < 0.05) between groups at the same time point (at corresponding developmental stage).

Our qPCR analyses showed that following vitrification-warming and in vitro maturation of mouse GV oocytes, the relative mRNA expressions of SAC-related genes in GV (*Mad1*, *BubR1*, and *Mad2)*, GVBD (*Mps1*, *Mad1*, *BubR1*, and *Mad2*), MI (*Mps1*, *BubR1*, and *Mad2*), and MII stages (*Mps1* and *Mad1*) were significantly (*p* < 0.05) downregulated in the vitrification group compared to the fresh (control) group (Figure 6). Conversely, addition of 10^−7^ mol/L MT in vitrification-warming and in vitro maturation media ameliorated the relative expressions of SAC-related genes in GV oocytes (*Mad1*, *BubR1*, and *Mad2*) and MII oocytes (*Mps1* and *Mad1*) in vitrification + MT group compared to the vitrification group (Figure 6).

## 4. Discussion

In recent years, assisted reproductive biotechnologies such as in vitro maturation and cryopreservation of oocytes have remained the center of attention for many researchers and clinicians working in the field of reproductive medicine. However, the search for developing a mutually beneficial procedure where both of these technologies could provide the optimal conditions for improved survival and development rates, and clinical outcomes, is crucial for forming the efficient treatment strategies [55]. Therefore, the quest for improvement in oocyte cryopreservation protocols still remains in the scope of cryobiologists. To date, many research groups including our lab [1,24] have demonstrated that oocyte cryopreservation could be successfully implemented for preserving mature oocytes. However, little is understood about the efficiency of combinatory application of cryopreservation and in vitro maturation of immature oocytes [56]. Therefore, in order to improve the efficiency and practical application of cryopreservation technology, in this study, we focused on the elucidation of potential mechanisms by which MT could ameliorate the developmental competence of vitrified-warmed mouse GV oocytes.

Oocyte development and maturation are very complex physiological processes which require the participation of many hormones and other biologically important factors [57]. Following vitrification, oocytes are subjected to severe damage, and consequently their developmental potential is substantially reduced [5]. The in vitro maturation rate of vitrified-warmed GV oocytes was significantly decreased in several animal models such as bovine [58,59], equine [60], porcine [61], sheep [62], and human [56]. Similar to the results of previous studies, in current study, we also observed that in vitro maturation rate of mouse GV oocytes was significantly reduced following vitrification-warming procedures. As observed in current study, this decline in development potential of GV oocyte is likely to be associated with an excessive ROS production, perturbations in mitochondrial membrane potential, impaired spindle configuration, and changes in the expression of essential regulatory genes following vitrification.

The decreased in vitro development of vitrified-warmed mouse oocytes could be improved by MT supplementation. For example, the 10^−4^ mol/L MT can improve the in vivo developmental potential of cumulus-oocyte-complex following cryopreservation of mouse ovaries and transplantation [63]. Recently, it was shown that 10^−11^ mol/L MT could ameliorate the in vitro survival rate of mouse preantral follicles following vitrification-warming [64]. In addition, we have demonstrated that 10^−9^ mol/L MT could improve the developmental potential of parthenogenetically activated embryos derived from vitrified mature oocytes [1,24]. Intriguingly, in this study, we screened the optimal concertation of MT at which an improved development competence of vitrified-warmed GV oocytes could be achieved. Our results showed that in vitro maturation rate of mouse GV oocytes was significantly increased following addition of 10^−7^ mol/L MT in vitrification-warming and in vitro maturation media. Therefore, it is reasonably understandable that these ameliorative effects of MT are concentration-dependent and could vary depending on stage of development of oocytes or embryos. Too high or too low doses of MT may be harmful for oocytes [43], subsequently diminishing their developmental competence. Whereas, at the optimal concentrations, MT could improve the in vitro development of vitrified-warmed oocytes, potentially by scavenging excessive ROS, ameliorating the redox status, stabilizing cellular organelles, and modulating the expression of important regulatory genes [1,24]. Although reports on effect of MT supplementation on developmental competence of vitrified GV oocytes are spare, in a recent report, Gao and colleagues [26] have demonstrated that supplementation of in vitro maturation media with MT (10^−11^ mol/L), could significantly improve the meiotic maturation (increased first polar body extrusion rate) of vitrified oocytes compared to the fresh control group. It should be noted that, in contrast to this report, MT (10^−7^ mol/L) was supplemented in all media used in vitrification-warming and in vitro maturation procedures carried out in our study.

Furthermore, we observed that following vitrification and in vitro maturation of mouse GV oocytes, GSH levels were significantly decreased, and ROS levels were significantly increased in GV, MI, and MII oocytes. After vitrification and warming, oocytes often showed an impaired GSH synthesis [65] and an excessive ROS production [66], leading to the decreased developmental potential of oocytes [65]. It has been suggested that adequate levels of GSH are essential for eliminating the excessive ROS produced during mitochondrial metabolism [67], maintaining the intracellular redox balance, and alleviating the vitrification-related oxidative stress [1,66]. In this study, following addition of 10^−7^ mol/L MT, GSH levels were significantly increased, ROS levels were decreased, and in vitro maturation rate of GV oocytes was significantly increased. On one hand, MT can directly scavenge ROS, which is consistent with most other antioxidants scavenging free radicals [68,69] and reducing the intracellular ROS levels. Besides, MT could also scavenge excessive ROS production and increases GSH production by modulating the mitochondrial function [70], and thereby promoting the in vitro development of oocytes. It has also been shown that MT (10^−6^) supplementation could ameliorate the nuclear and cytoplasmic maturation of in vitro matured mouse oocytes by decreasing ROS production and increasing GSH levels [71].

In this study, the mitochondrial membrane potential in GV, MI, and MII oocytes was significantly decreased following vitrification; similarly, ATP levels in MII oocytes were also significantly decreased. It is widely believed that mitochondria are the essential cellular organelles linked to energy production and supply in cells [72]. The high mitochondrial membrane potential is a prerequisite for ATP production [26,73]; the optimal mitochondrial function and ATP levels could promote the cytoplasmic maturation of oocytes and development of embryos in vitro [59]. Therefore, any damage to mitochondria or changes in the mitochondrial function might perturb the normal oocyte development. The decreased mitochondrial activity due to oocyte cryopreservation in this study and previous reports [23,74] changed the mitochondrial membrane potential, potentially leading to the decreased oxidative phosphorylation [75] and ATP concentrations [25,72], which subsequently impact the oocyte maturation and embryo development in vitro [23,26,72,74]. However, in this study, MT supplementation (10^−7^ mol/L) significantly improved the mitochondrial function in vitrified-warmed mouse oocytes, and the ATP levels were also increased in resultant MII oocytes, potentially leading to the improved in vitro development. Similar results were also obtained in the vitrified-warmed bovine mature oocytes, where addition 10^−9^ mol/L MT in vitrification media improved their parthenogenetic development [31], and in vitrified mouse GV oocytes, which demonstrated that 10^−11^ mol/L MT in in vitro maturation media showed a positive effect on their in vitro development [26]. It seems that these ameliorative effects of MT on mitochondrial function may be achieved by reducing the ROS levels [26,70,76].

In this study, normal morphology of spindles in MII oocytes (matured from vitrified GV oocytes) was significantly decreased following vitrification-warming of mouse GV oocytes. It is believed that low temperature is an important factor linked to the spindle assembly, and could potentially perturb the polymerization of microfilaments [27,77]. Even if the oocytes were treated at the transient cooling to room temperature, it could disrupt the normal spindle assembly [78]; the incidence of abnormal spindle could significantly increases following slow controlled-rate freezing [79]. This may be attributed to the decreased mitochondrial membrane potential and subsequently reduced ATP content in oocytes following cryopreservation and in vitro culture [26], leading to an insufficient provision of energy for meiotic spindle assembly and maintenance [80]. However, as expected, addition of MT (10^−7^ mol/L) in vitrification-warming and in vitro maturation media improved the normal spindle morphology in oocytes. Previously, it has been shown that MT could increase the ATP content [26,31], facilitate the spindle assembly, and reduce the incidence of abnormal spindle morphology [26,81], thereby promoting the in vitro meiotic maturation of oocytes [26]. It has also been suggested that, following MT supplementation, an increased number of mtDNA copies in vitrified oocytes may be a potential means for supporting the balance of mitochondrial function and chromosomal alignment [26].

Cryopreservation has been implicated in causing alterations in the relative expression of key regulatory genes in oocytes [1,24,82]. Following vitrification-warming of mouse GV oocytes, the relative expressions of *Grp78* and *Chop* were significantly increased after 24 h in vitro culture [83]. Besides, in our previous report, when bovine GV oocytes were vitrified-warmed, the relative expression of *CD9* was significantly decreased, and the relative expression of *CD81* was significantly increased after 1–2 h in vitro culture [58]. Similarly, when bovine immature oocytes were vitrified-warmed and in vitro cultured for 24 h, the relative expression of *CDC20* was significantly decreased, and the relative expression of *Eg5* and *P53* was significantly increased [84]. Moreover, following vitrification-warming of MII oocytes, the relative expressions of *Dcp1a*, *Dcp2*, and *Pou5f1* in mouse oocytes [24], and *CD9* gene in bovine oocytes were significantly decreased [82], meanwhile the relative expression of *mn-Sod* was increased in mouse oocytes [85]. Intriguingly, in this study the relative expressions of SAC-related genes—i.e., *BubR1*, *Mad1*, and *Mad2*—were significantly decreased after vitrification-warming of mouse GV oocytes. Of note, the relative expression of *Mad1* and *Mps1* was significantly decreased after 12–14 h in vitro culture of vitrified-warmed mouse GV oocytes. It is believed that SAC-related genes and proteins play essential regulatory roles in normal spindle assembly and chromosome segregation during meiosis [34], in particular, Mad1 and Mps1 play important roles in spindle assembly [34,37], and their normal expression is critical for chromosome separation and correct attachment to the kinetic microtubules [34]. Perturbation in the expression of these genes lead to the alterations in expression of key SAC-related proteins, and consequent abnormalities in spindle assembly could affect oocyte meiosis, leading to the incorrect and impaired separation of sister chromosomes, and as a result the developmental competence of oocytes is further reduced [37,38]. Interestingly, when MT (10^−7^ mol/L) was added in vitrification-warming and in vitro maturation media used in this study, the relative expressions of *Mps1* and *Mad1* were significantly increased in mature oocytes developed from vitrified-warmed GV oocytes and returned to levels similar to the control group. These findings, in corroboration with the improved developmental rates of vitrified-warmed GV oocytes treated with MT, reasonably indicate that improved mRNA expression of SAC-related genes might result in the recovery of normal expression of corresponding proteins, and hence could rescue the morphological abnormalities in spindles, leading to the normal separation of sister chromosomes, and subsequent significantly improved developmental rates compared to the untreated vitrified oocytes. Nevertheless, further mechanistic studies are still awaited to improve our understanding in this regard.

Intriguingly, in addition to the enticing findings of this study, recently a few reports (reviewed in [86]) have demonstrated that MT could also produce its ameliorative effects during in vitro oocyte maturation and can improve embryo development through its membrane bound receptors (MT_1_ or MT_2_). This notion is further supported by the findings of Casao and colleagues [87] who reported that the addition of MT receptor antagonists to the IVM medium did not affect the maturation, fertilization, or cleavage rates, but decreased the blastocyst rate in ovine model. Therefore, it would be interesting to conduct future studies aimed at evaluating similar receptor-mediated effects of MT on in vitro developmental potential of vitrified-warmed mammalian oocytes, in particular at the GV stage.

## 5. Conclusions

To sum up, vitrification of mouse GV oocytes leads to the decreased mitochondrial membrane potential and ATP content, increased ROS levels, decreased GSH levels, morphological anomalies in spindles, and altered the mRNA expression of SAC-related genes (*BubR1*, *Mad1*, *Mad2*, *Mps1*) during in vitro maturation, and consequently reduced the in vitro maturation of GV oocytes. However, following addition of 10^−7^ mol/L MT throughout the whole processes of vitrification-warming and in vitro maturation, redox homeostasis (ROS/GSH) was ameliorated, and in vitro development of vitrified-warmed mouse GV oocytes was improved, potentially by regulating the mitochondrial function, spindle morphology, and the expression of SAC-related genes (*BubR1*, *Mad1*, *Mad2*, *Mps1*). These findings add great value to our understanding of potential mechanism by which MT might ameliorate the developmental competence of vitrified-warmed GV oocytes, and provide a reasonable theoretical reference for further improving the cryopreservation technology focused on immature mammalian oocytes.

## Figures and Tables

**Table 1:** PCR primers used for SYBR green Q-PCR analysis.

Gene	Accession ID	Primer Seq (5′-3′)	Product Length (bp)	Tm (°C)
*Mad2*	NM_001355624.1	F: GAGAGCAAGGCATCACCCTG	103	60.2
		R: TCCGACGGATAAATGCCACG		
*BubR1*	NM_009773.3	F: TCCAGGAAGGGATTGAACGC	154	60.2
		R: AGCGAGCTTCTCTGTGGTTC		
*Mps1*	NM_001110265.1	F: CCATGGGAACGCAAGAGCTA	284	60.2
		R: GCTCTTCCCGGTACCTTGGTC		
*Mad1*	NM_001359025.1	F: CCTGGGATCCTACGACAGTG	105	60.2
		R: CTGTGGGCATGTACCTTCTGA		
*G* *apdh*	NM_008084.3	F: CATGGCCTTCCGTGTTCCTA	104	60.2
		R:GCCTGCTTACCACCTTCTT		

**Table 2:** In vitro maturation of vitrified mouse GV oocytes after melatonin treatment.

Groups	Treated with Melatonin(mol/L)	No. of GV Oocytes Vitrified	No. of GV Oocytes Recovered	No. of GV Oocytes with Normal Morphology	No. of GV Oocytes that Matured to		
					GVBD Stage at 5 h.p.i. (%)	MI Stage at 9 h.p.i. (%)	MII Stage at 13 h.p.i. (%)
F	0			54	51(95.45 ± 0.04) ^a^	47(85.76 ± 0.05) ^a^	44(81.21 ± 0.01) ^a^
V	0	67	60	56	46(85.93 ± 0.06) ^b,e^	41(74.07 ± 0.04) ^b,e,f^	35(61.30 ± 0.02) ^c^
V + M	10^−9^	65	59	57	26(45.71 ± 0.01) ^c^	19(34.09 ± 0.05) ^c^	11(19.39 ± 0.01) ^f^
V + M	10^−7^	65	61	52	47(89.97 ± 0.04) ^a,b^	41(78.52 ± 0.06) ^a,e^	38(72.67 ± 0.06) ^b^
V + M	10^−5^	63	56	52	41(78.75 ± 0.03) ^e^	37(71.25 ± 0.03) ^b,f^	20(37.92 ± 0.06) ^d^
V + M	10^−3^	62	56	50	30(59.98 ± 0.01) ^d^	26(52.09 ± 0.02) ^d^	15(29.80 ± 0.03) ^e^

The fresh GV oocytes were randomly divided into three groups: untreated (control, F), or vitrified by open-pulled straw method without (vitrification group, V) or with melatonin (MT) supplementation (vitrification + MT group, V + M). In vitrification + MT groups, MT was added to all media at final concentrations of 10^−9^, 10^−7^, 10^−5^, and 10^−3^ mol/L, respectively in the entire experimental procedures. Morphologically normal oocytes were evaluated by visual inspection of membrane integrity, zona pellucida (ZP), and any altered appearance of cytoplasm (e.g., becoming white, colorless, or dispersed). The percentage of GVBD oocytes, MI oocytes and MII oocytes were calculated from the number of oocytes with normal morphology. Values with different superscripts (a, b, c, d, e and f) in the same column differ significantly (*p* < 0.05). Notes: GV: germinal vesicle; GVBD: germinal vesicle breakdown; MI: metaphase I; MII: metaphase II; h.p.i: hours post in-vitro culture.
